# OP31 Monitoring Of The Budget Impact Determinants Of Incorporated Technologies For Rare Diseases In The Brazilian Health System

**DOI:** 10.1017/S026646232400093X

**Published:** 2025-01-07

**Authors:** Mariá Gonçalves Pereira Silva, Amanda Oliveira Lyrio, Laís Lessa Neiva Pantuzza, Thais Conceição Borges, Barbara Pozzi Ottavio, Ana Carolina de Freitas Lopes, Luciene Bonan

## Abstract

**Introduction:**

Budget impact analyses for the treatment of rare diseases are especially important for the sustainability of health systems due to high treatment costs and uncertainties in target population estimates. The objective of this work is to analyze the elements that influence discrepancies between predicted and observed budget impacts for enzyme replacement therapies for rare diseases in Brazil’s public health system.

**Methods:**

All enzyme replacement therapies for rare diseases evaluated by the National Committee for Health Technology Incorporation in the Brazilian Public Health System (Conitec) and with at least one year of use were included. For each technology, the following were identified: number of patients, median patient weight, annual quantity of medication, unit price, and budget impact. The attributes were compared between previous estimates and real-world observation after use. The data sources were publicly accessible administrative databases and Conitec technical reports.

**Results:**

Five technologies were selected: elosulfase alfa, alglucosidase alfa, idursulfase, laronidase, and galsulfase. In the first year, the difference between the estimated and the observed number of patients treated was up to 15 percent lower or higher for four technologies, but with monthly fluctuation throughout the year. The median weight of users was between 23 percent and 468 percent higher for three technologies. The observed price was as expected, with variations between three percent lower and 14 percent higher. The quantity of medicines used was lower (between 39% and 46%) than expected for all technologies. The observed budget impact was 37 percent to 47 percent lower than estimated.

**Conclusions:**

Real-world budget impact was lower than expected for all technologies. The main cause of discrepancies was the estimate of the annual amount of medication, which did not consider gradual adherence and discontinuation of treatment. This highlights the need to review the budget impact methodology for rare diseases, forecasting monthly market share and treatment discontinuation rate.

